# The Hawaiian freshwater algae biodiversity survey (2009–2014): systematic and biogeographic trends with an emphasis on the macroalgae

**DOI:** 10.1186/s12898-014-0028-2

**Published:** 2014-10-25

**Authors:** Alison R Sherwood, Amy L Carlile, Jessica M Neumann, J Patrick Kociolek, Jeffrey R Johansen, Rex L Lowe, Kimberly Y Conklin, Gernot G Presting

**Affiliations:** 1 Department of Botany, University of Hawaii, 3190 Maile Way, Honolulu 96822, HI, USA; 2 Current address: Department of Biology and Environmental Science, University of New Haven, 300 Boston Post Road, West Haven 06516, CT, USA; 3 Department of Ecology and Evolutionary Biology and Museum of Natural History, University of Colorado, Boulder 80309, CO, USA; 4 Department of Biology, John Carroll University, University Heights 44118, OH, USA; 5 Department of Biological Sciences, Bowling Green State University, Bowling Green 43409, OH, USA; 6 Department of Molecular Biosciences and Bioengineering, University of Hawaii, 1955 East-West Road, Honolulu 96822, HI, USA

**Keywords:** Algal distribution, Biodiversity survey, Biogeography, Cyanobacteria, Dispersal, Freshwater algae, Hawaiian Islands, Molecular characterization, Taxonomy, UPA

## Abstract

**Background:**

A remarkable range of environmental conditions is present in the Hawaiian Islands due to their gradients of elevation, rainfall and island age. Despite being well known as a location for the study of evolutionary processes and island biogeography, little is known about the composition of the non-marine algal flora of the archipelago, its degree of endemism, or affinities with other floras. We conducted a biodiversity survey of the non-marine macroalgae of the six largest main Hawaiian Islands using molecular and microscopic assessment techniques. We aimed to evaluate whether endemism or cosmopolitanism better explain freshwater algal distribution patterns, and provide a baseline data set for monitoring future biodiversity changes in the Hawaiian Islands.

**Results:**

1,786 aquatic and terrestrial habitats and 1,407 distinct collections of non-marine macroalgae were collected from the islands of Kauai, Oahu, Molokai, Maui, Lanai and Hawaii from the years 2009–2014. Targeted habitats included streams, wet walls, high elevation bogs, taro fields, ditches and flumes, lakes/reservoirs, cave walls and terrestrial areas. Sites that lacked freshwater macroalgae were typically terrestrial or wet wall habitats that were sampled for diatoms and other microalgae. Approximately 50% of the identifications were of green algae, with lesser proportions of diatoms, red algae, cyanobacteria, xanthophytes and euglenoids. 898 DNA sequences were generated representing eight different markers, which enabled an assessment of the number of taxonomic entities for genera collected as part of the survey. Forty-four well-characterized taxa were assessed for global distribution patterns. This analysis revealed no clear biogeographic affinities of the flora, with 27.3% characterized as “cosmopolitan”, 11.4% “endemic”, and 61.3% as intermediate.

**Conclusions:**

The Hawaiian freshwater algal biodiversity survey represents the first comprehensive effort to characterize the non-marine algae of a tropical region in the world using both morphological and molecular tools. Survey data were entered in the Hawaiian Freshwater Algal Database, which serves as a digital repository of photographs and micrographs, georeferenced localities and DNA sequence data. These analyses yielded an updated checklist of the non-marine macroalgae of the Hawaiian Islands, and revealed varied biogeographic affinities of the flora that are likely a product of both natural and anthropogenic dispersal.

## Background

Eight main islands and 124 small islands, atolls and shoals comprise the Hawaiian Archipelago, which encompasses 16,640 km^2^ and represents the largest oceanic archipelago in the world [1],[2]. The islands are volcanically-derived, and form successively as the Pacific Plate moves in a northwestern direction over a fixed “hot spot”, such that the eight current high islands (The Main Hawaiian Islands) at the southeastern end of the chain represent only the last 5.1 million years (my) of a 75–80 my history of volcanic activity [3]. The Main Hawaiian Islands are characterized by steep gradients of elevation (0–4,200 m), rainfall (25–1,050 cm/year) and island age (0–5.1 my), which have generated a remarkable range of ecological conditions [2],[4].

The Hawaiian Islands are well known as a uniquely isolated and time-calibrated environment in which to study evolution; a place where numerous lineages of plants and animals exemplify the phenomena of endemism and adaptive radiation [5]-[9]. The geographical isolation of the Main Hawaiian Islands (>3,500 km from the nearest continent) is a strong force influencing the biogeography of Hawaii, and initial colonization of the islands was accomplished through the arrival and survival of dispersed individuals [1]. Subsequent colonization likely occurred through a combination of long-distance dispersal, and, increasingly commonly, dispersal from adjacent islands [3].

A number of studies on the freshwater algae of the Hawaiian Islands have appeared in the literature [10]-[20]. Eight hundred taxonomic records were compiled from the literature in a bibliographic checklist spanning the years 1876–2003 [21], which included representatives of almost all major freshwater algal lineages. Based on these literature records (which are almost exclusively based on morphospecies identifications), the overall level of endemism for the Hawaiian non-marine algae was estimated to be 5%, or a total of 40 taxa. This endemism level was noted as being very low in comparison to other organisms such as marine red algae (20%), marine invertebrates (32%), ferns and lycophytes (74%), flowering plants (79%), and insects (94%) [4],[9],[22]. It was subsequently noted that the stream algal flora of Hawaii was “suspiciously cosmopolitan” in composition [23], drawing attention to the large proportion of Hawaiian taxonomic records in common with very different biogeographic regions of the world. Others have noted that freshwater algae around the world have often been considered to be cosmopolitan, that this notion is unlikely to hold true when tested with genetic data, and that endemism is most likely obscured by the “force-fitting” of European names [24].

Competing explanations exist for the biogeographic distributions of freshwater algae. Distributions of microbial algae can be examined in the context of “*Everything is everywhere*, but, *the environment selects*” [25], which predicts that microorganisms are globally distributed as a result of their vast population sizes and unlimited dispersal, and whether or not a species becomes established is determined solely by local conditions [26],[27]. In contrast, a number of researchers have reported evidence of biogeographic patterning of microorganisms by using larger sample sizes, detailed observations, and/or molecular assessments of diversity [28]-[32], and still others invoke a combination of ubiquity and endemism to explain cryptic molecular lineages of widespread morphospecies [33],[34]. Whether Hawaiian freshwater algae are truly cosmopolitan when molecular comparisons are taken into account, or whether they display levels of endemism that parallel other elements of the flora and fauna of the archipelago, is currently unknown.

Given current estimates of biodiversity loss, it is critical to understand the scope of endemism versus cosmopolitanism in the poorly characterized freshwater flora of a region already recognized as a biodiversity hotspot for other lineages of organisms. Loss of biodiversity is proceeding faster in freshwaters than in any other major biome [35]-[37]. The Hawaiian Freshwater Algal Biodiversity Survey (2009–2014) was carried out to establish baseline data to further assess human impacts on freshwater Hawaiian ecosystems and monitor change in the context of biodiversity conservation. The goals of the survey were to collect, document, and characterize as many non-marine algal taxa as possible from the Main Hawaiian Islands, and to use the resultant data to examine distribution patterns and develop biogeographic hypotheses to explain these patterns. The first author (ARS) held responsibility for the characterization of the macroalgal samples resulting from the survey, and as such the focus of the present report is on these taxa. Expeditionary collections were made on the islands of Kauai, Oahu, Molokai, Lanai, Maui and Hawaii, and macroalgal specimens were characterized morphologically and, where possible, using molecular methods. The distributional patterns and biogeographic affinities of taxa based on both literature records and DNA sequence analyses were examined in the context of distributions characterized by cosmopolitanism (as defined by reported presence in most other regions of the world) versus endemism (as defined by a lack of records outside the Hawaiian Islands). With very little information currently available for biogeographic patterns of specific freshwater algae based on molecular data trends, no predictions of biogeographic affinity were made for the Hawaiian non-marine algae. However, an increase in the number of recognized endemic taxa was predicted, given the frequency with which freshwater algae are demonstrated to harbor cryptic (only revealed at the molecular level) diversity or pseudocryptic (not immediately evident at the morphological level but in retrospect discernable once the species boundaries are highlighted with molecular data) diversity when molecular tools are employed.

## Results

### Summary of collections

**Figure 1:**
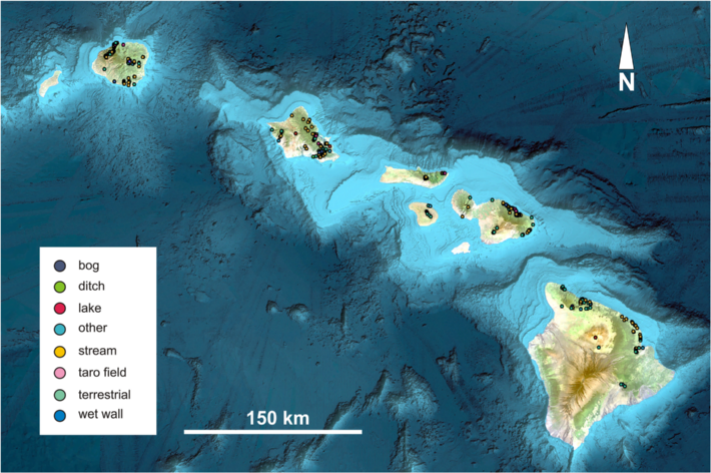
**Map of collection sites.** The 1,786 locations, or “environmental accessions” sampled as part of the Hawaiian Freshwater Algal Biodiversity Survey. Habitat types are coded by color (legend on figure).

**Figure 2:**
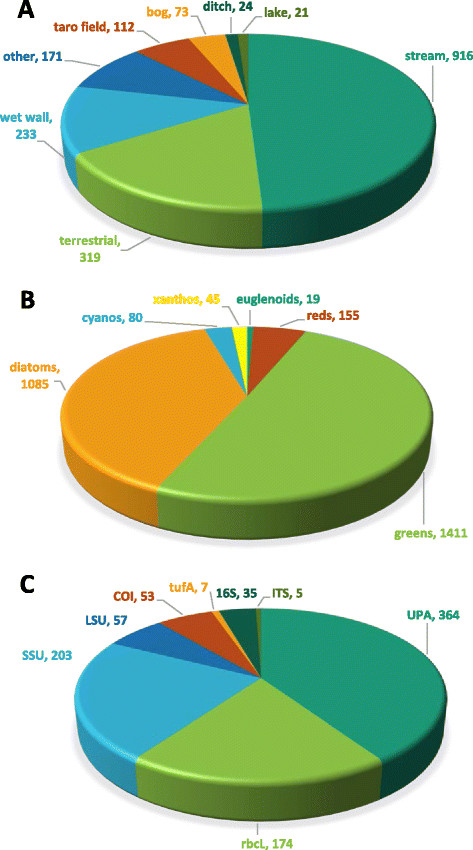
**Summary statistics for the Hawaiian freshwater algal biodiversity survey.** Summary statistics from the biodiversity survey. **A)** proportion and number of total environmental accessions represented by each habitat type, **B)** proportion and number of total isolate accessions represented by each major algal lineage, **C)** proportion and number of total DNA sequences generated represented by each molecular marker.

A total of 1,786 distinct sites were sampled (“environmental accessions”) (Figure 1). Collection sites were concentrated on the wetter, windward sides of the islands, and on the larger islands that had a greater number of perennial streams and associated moist habitats. Sampling sites were not chosen randomly, but were selected on the basis of accessibility and likelihood of harboring interesting algal diversity; thus, the following numbers should be interpreted as a summary of the characteristics of the collections rather than an indication of available habitats in the Hawaiian Islands. Areas not sampled lacked suitable habitats or were not accessible. Streams comprised 49.0% of all sites, followed by terrestrial sites (17.1%) and wet walls (12.5%), with smaller proportions of the remaining habitat categories (Figure 2a). A total of 2,823 isolate accessions (i.e. separate algal identifications) were characterized, of which 1,407 were macroalgae. Slightly more than 50.0% of these isolate accessions were green algae, while diatoms (38.8%), red algae (5.5%), cyanobacteria (2.9%), xanthophytes (1.6%) and euglenoids (0.7%) comprised the remainder (Figure 2b). Additional file 1 summarizes the taxa identified, along with their islands of distribution and the habitat types from which they were collected and identified. The most widely collected taxa included the green algae *Spirogyra* spp. (300 accessions), *Mougeotia* spp. (144 accessions), *Oedogonium* spp. (102 accessions), *Cladophora glomerata* (89 accessions), *Microspora* spp. (78 accessions), *Cloniophora spicata* (69 accessions), and *Rhizoclonium* spp. (64 accessions), the red algal species *Compsopogon caeruleus* (34 accessions) and the xanthophyte *Vaucheria* spp. (31 accessions).

### DNA barcoding

A total of 898 DNA sequences were generated as part of the survey, representing eight different markers (Figure 2c) [GenBank accessions for those sequences not previously published: KM676560 - KM676564 for ITS, KM676565 - KM676567 for *tuf*A, KM676568 - KM676828 for UPA, KM676829 - KM676881 for LSU, KM676882 - KM677026 for SSU, KM677027 - KM677127 for *rbcL*]. The UPA marker, which was employed as an initial screen for all samples, accounted for more sequences than any other marker (364 total), while SSU (203), *rbcL* (174), LSU (57), COI (53) and 16S rRNA (35) comprised the majority of the remainder.

**Figure 3:**
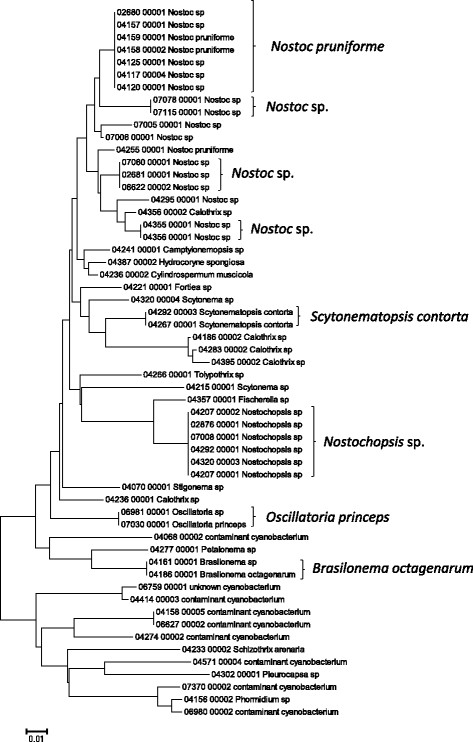
**Neighbor-joining tree of UPA sequences of the Hawaiian non-marine cyanobacteria.** Sequence diversity based on the UPA marker for Hawaiian cyanobacterial specimens. The neighbor-joining tree is based on uncorrected p-distances nucleotide model in MEGA 5.05. Scale bar = substitutions per site.

**Figure 4:**
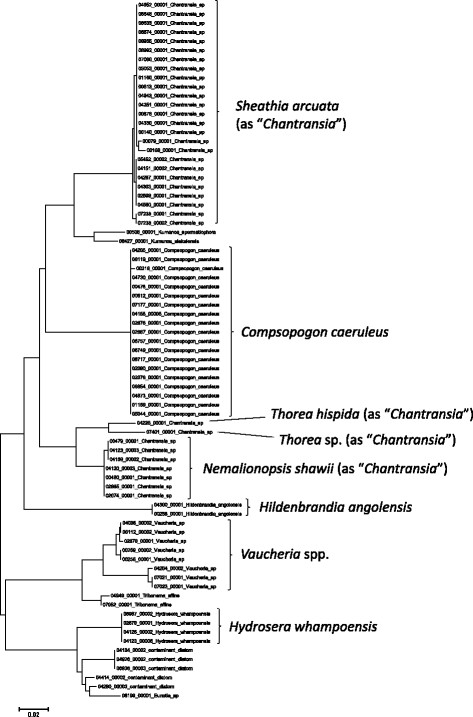
**Neighbor-joining tree of UPA sequences of the Hawaiian non-marine red algae, diatoms and xanthophyte algae.** Sequence diversity based on the UPA marker for the Hawaiian non-marine red algae, diatoms and xanthophytes. The neighbor-joining tree is based on uncorrected p-distances nucleotide model in MEGA 5.05. Scale bar = substitutions per site.

**Figure 5:**
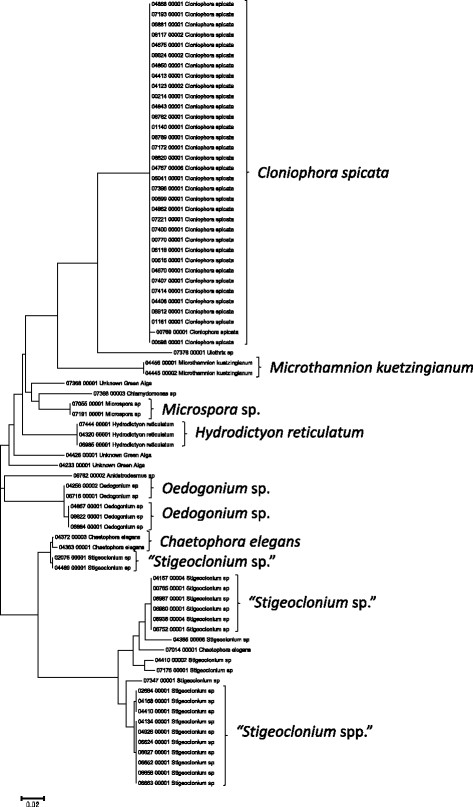
**Neighbor-joining tree of UPA sequences of the Hawaiian non-marine, non-charophycean green algae.** Sequence diversity based on the UPA marker for the Hawaiian non-marine, non-charophycean green algae. The neighbor-joining tree is based on uncorrected p-distances nucleotide model in MEGA 5.05. Scale bar = substitutions per site.

**Figure 6:**
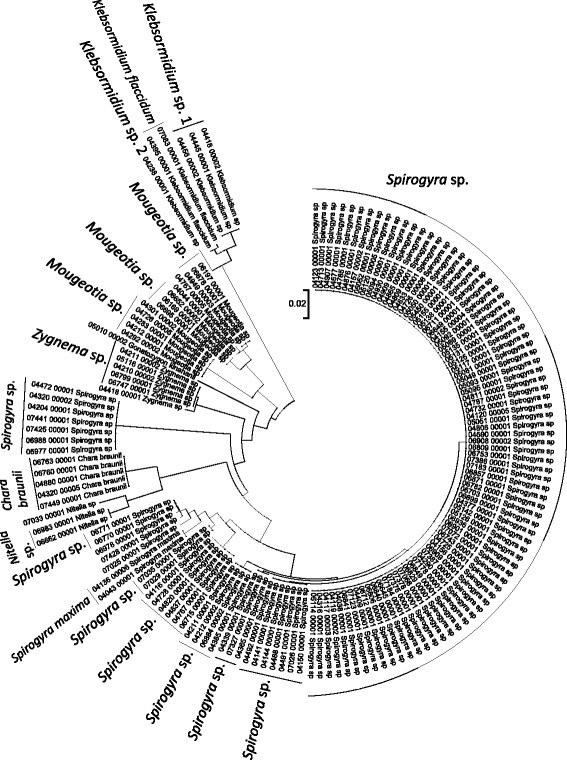
**Neighbor-joining tree of UPA sequences of the Hawaiian non-marine charophycean green algae.** Sequence diversity based on the UPA marker for the Hawaiian non-marine, charophycean green algae. The neighbor-joining tree is based on uncorrected p-distances nucleotide model in MEGA 5.05. Scale bar = substitutions per site.

**Figure 7:**
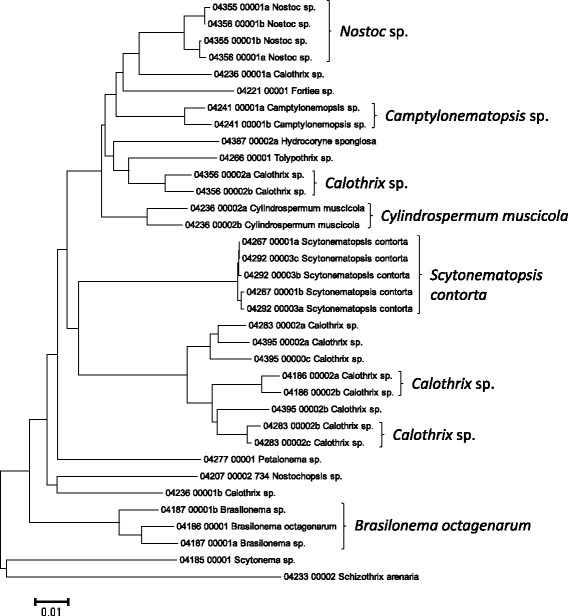
**Neighbor-joining tree of 16S rRNA gene sequences of the Hawaiian non-marine cyanobacteria.** Sequence diversity based on the 16S rRNA marker for the Hawaiian non-marine cyanobacteria. The suffixes “a”, “b” or “c” after an accession name indicate clone isolates of that sample. The neighbor-joining tree is based on uncorrected p-distances nucleotide model in MEGA 5.05. Scale bar = substitutions per site.

**Figure 8:**
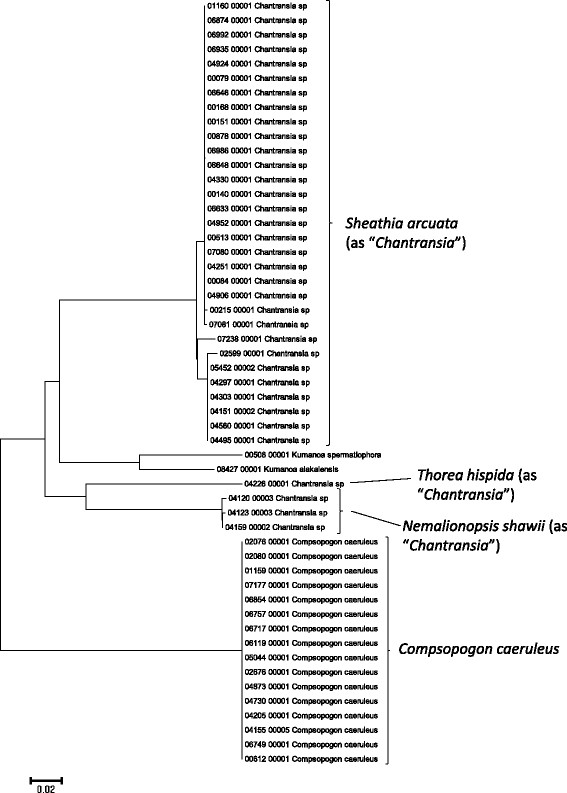
**Neighbor-joining tree of COI sequences of the Hawaiian non-marine red algae.** Sequence diversity based on the COI marker for the Hawaiian non-marine red algae. The neighbor-joining tree is based on uncorrected p-distances nucleotide model in MEGA 5.05. Scale bar = substitutions per site.

**Figure 9:**
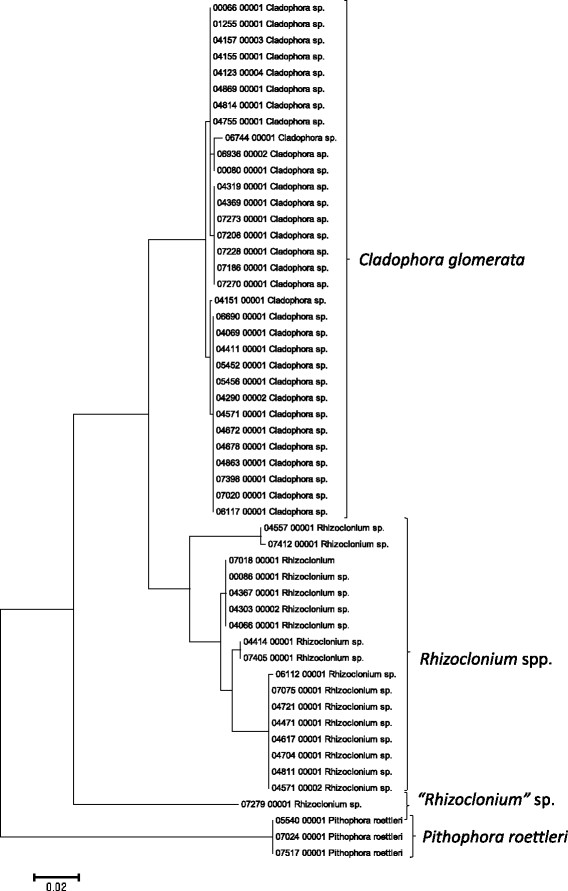
**Neighbor-joining tree of LSU sequences of the Hawaiian non-marine, non-charophycean green algae.** Sequence diversity based on the LSU marker for the Hawaiian non-marine, non-charophycean green algae. The neighbor-joining tree is based on uncorrected p-distances nucleotide model in MEGA 5.05. Scale bar = substitutions per site.

**Figure 10:**
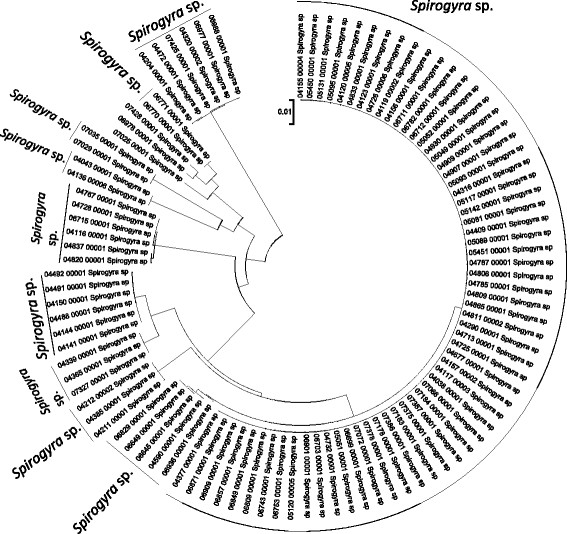
**Neighbor-joining tree of** ***rbcL*** **sequences of the Hawaiian non-marine charophycean green algae.** Sequence diversity based on the *rbcL* marker for the Hawaiian non-marine charophycean green algae. The neighbor-joining tree is based on uncorrected p-distances nucleotide model in MEGA 5.05. Scale bar = substitutions per site.

**Figure 11:**
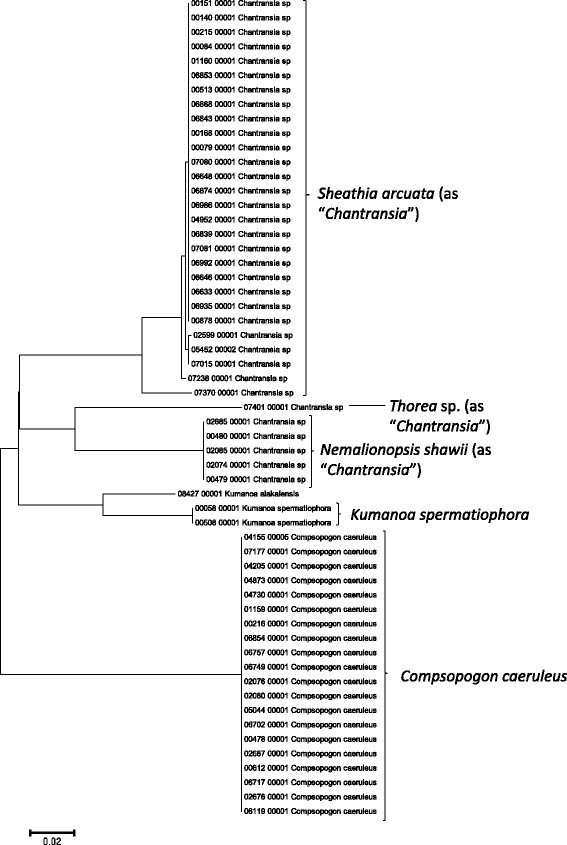
**Neighbor-joining tree of** ***rbcL*** **sequences of the Hawaiian non-marine red algae.** Sequence diversity based on the *rbcL* marker for the Hawaiian non-marine red algae. The neighbor-joining tree is based on uncorrected p-distances nucleotide model in MEGA 5.05. Scale bar = substitutions per site.

**Figure 12:**
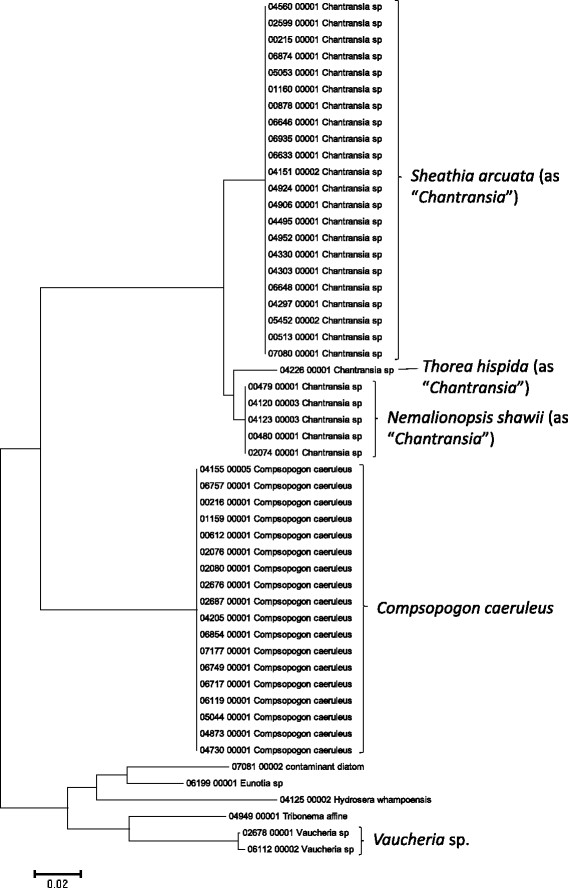
**Neighbor-joining tree of SSU sequences of the Hawaiian non-marine red algae, diatoms and xanthophyte algae.** Sequence diversity based on the SSU marker for the Hawaiian non-marine red algae, diatoms and xanthophyte algae. The neighbor-joining tree is based on uncorrected p-distances nucleotide model in MEGA 5.05. Scale bar = substitutions per site.

**Figure 13:**
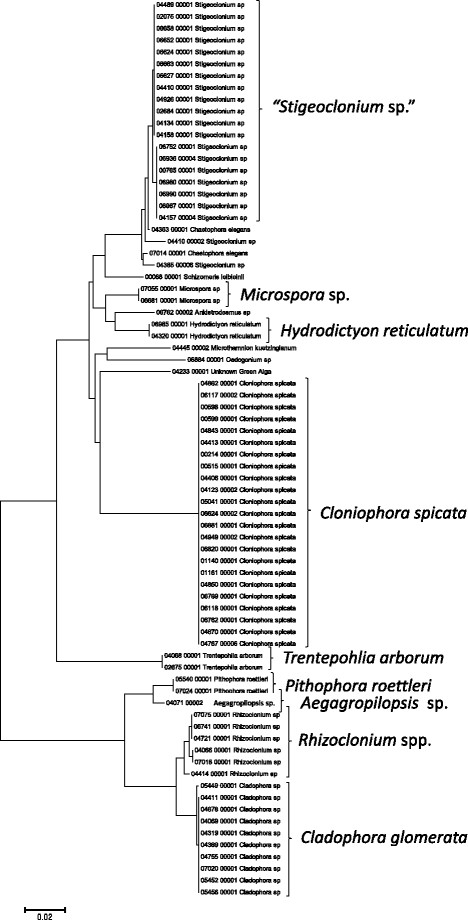
**Neighbor-joining tree of SSU sequences of the Hawaiian non-marine, non-charophycean green algae.** Sequence diversity based on the SSU marker for the Hawaiian non-marine, non-charophycean green algae. The neighbor-joining tree is based on uncorrected p-distances nucleotide model in MEGA 5.05. Scale bar = substitutions per site.

**Figure 14:**
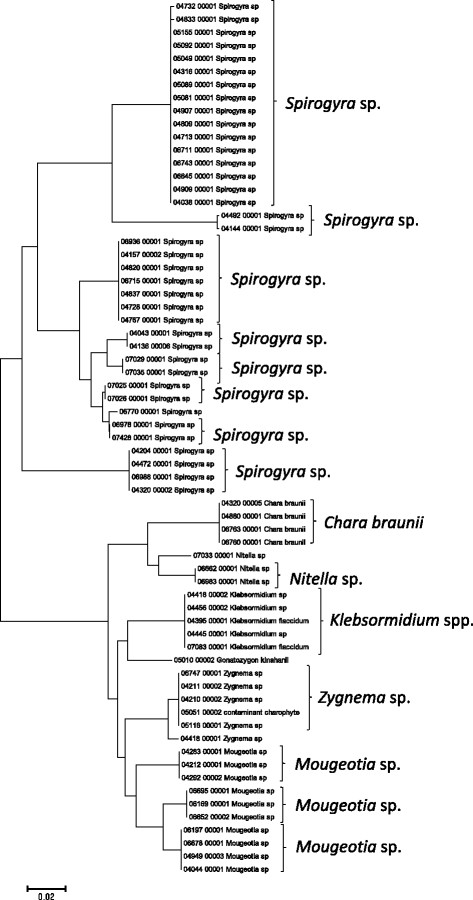
**Neighbor-joining tree of SSU sequences of the Hawaiian non-marine, charophycean green algae.** Sequence diversity based on the SSU marker for the Hawaiian non-marine charophycean green algae. The neighbor-joining tree is based on uncorrected p-distances nucleotide model in MEGA 5.05. Scale bar = substitutions per site.

Sequence data for the 16S rRNA, COI, LSU, SSU, *rbcL*, and UPA markers were employed to investigate the molecular diversity of specific lineages of the Hawaiian non-marine algal flora. Sequence diversity for each sampled taxon is represented in neighbor-joining trees based on uncorrected p-distances (Figures 3, 4, 5, 6, 7, 8, 9, 10, 11, 12, 13 and 14). Rather than attempting to reconstruct phylogenetic relationships for each taxon, we aimed to use these data for an assessment of patterns of molecular diversity within the Hawaiian flora. Thus, higher order relationships are not inferred from these analyses; comparisons should be restricted to closely-related taxa. More in-depth phylogenetic analyses have been published or are in progress for a number of taxa, including green and red algae [38],[39], cyanobacteria [40] and diatoms [41].

Summary taxon labels on each tree reflect the lowest taxonomic level to which a confident assignment can be made for a cluster of identical or near-identical sequences (i.e. those for which only single nucleotide differences were found, with the exception of those taxa known to harbor greater diversity, such as *Sheathia arcuata*). The vast majority of samples were not reproductive at the time of collection, which limited the number of taxa that could be identified to the species level. Nonetheless, clustering patterns of closely related sequences can be used as an indication of the number of entities represented in the Hawaiian flora for a given genus (at least for the commonly encountered entities). For example, the UPA analysis of cyanobacteria suggests eight species of the genus *Nostoc* were collected and characterized during the surveys (Figure 3), two of which were included in the 16S rRNA analysis (Figure 7). Four species of the red algal form genus “*Chantransia*” were identified from the UPA analysis, representing members of the Batrachospermales and Thoreales (Figure 4) [39], and these species-level groupings were consistent with groups recovered in the COI (Figure 8), *rbcL* (Figure 11) and SSU (Figure 12) analyses. The most species-rich genus of freshwater macroalgae studied in this survey was the charophycean green alga *Spirogyra*, which was demonstrated to consist of 12 clusters of sequences for the UPA marker (Figure 6), 13 for *rbcL* (Figure 10), and nine for SSU (Figure 14); an in-depth comparison of these sequences to accessions worldwide and a comparative analysis of thallus morphology is underway (J. Neumann and A. Sherwood, personal observations).

### Categories of distribution

**Figure 15:**
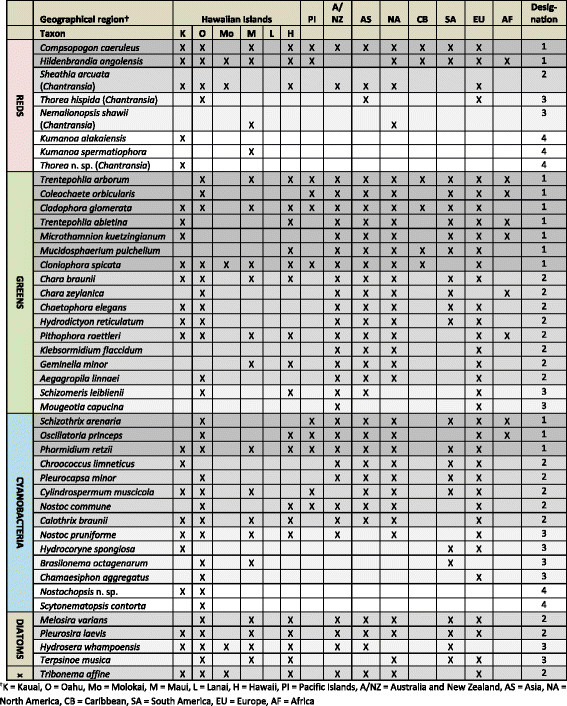
**Distributional analysis of well-characterized Hawaiian taxa representing the red algae, green algae, cyanobacteria, diatoms and xanthophytes.** Distribution on each of the Hawaiian Islands as determined by this survey is indicated, along with literature records of distribution from other regions of the world. Categories of distribution are as follows: 1 = reported from 6–8 regions outside of Hawaii, 2 = reported from 4–5 regions outside of Hawaii, 3 = reported from 1–3 regions outside of Hawaii, 4 = putatively endemic to the Hawaiian Islands. “X” = Xanthophytes. Taxon names are listed within each major group by distribution category, and alphabetically by taxon name.

The best-studied and best-represented taxa from the survey were examined for distributional trends: eight red algae, 17 green algae, 14 cyanobacteria, four diatoms and one xanthophyte, for a total of 44 taxa (Figure 15). Twelve taxa were determined to be in the category of broadest distribution (mostly green algae, but also including some red algae and cyanobacteria), 17 were in the category of second most broadly distributed (all groups except xanthophytes), 10 were members of the third category (all groups except xanthophytes), and five were members of the putatively endemic category (three red algae and two cyanobacteria). It should be noted, however, that sampling effort has not been consistent among these regions of the world, with some having received many times the effort (e.g. Europe, North America) than others (e.g. Africa, Pacific Islands) over the course of the past several centuries.

**Figure 16:**
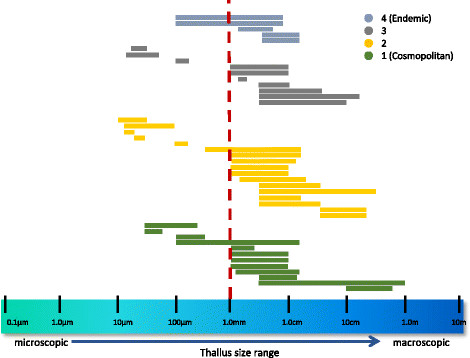
**Graphical representation of thallus size by category of distribution.** Graphical representation of thallus sizes of well-characterized Hawaiian non-marine algae, separated by category of distribution (as given for Figure 15).

**Figure 17:**
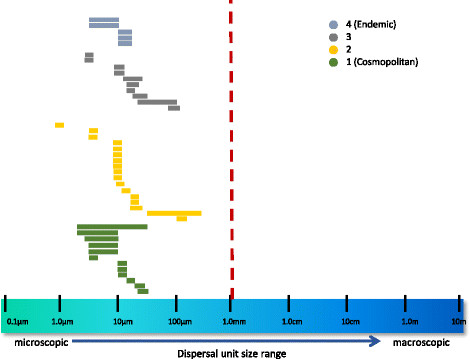
**Graphical representation of dispersal-unit sizes by category of distribution.** Graphical representation of dispersal-unit sizes of well-characterized Hawaiian non-marine algae, separated by category of distribution (as given for Figure 15).

For each of the taxa in each of the categories above, thallus size and dispersal unit size ranges (as determined from the literature) were plotted to assess patterns associated with dispersal category. Thallus sizes were found to range from microscopic to macroscopic for representatives of all dispersal categories (although not necessarily for all individual taxa) (Figure 16). In contrast, dispersal unit size ranges were found to be uniformly ≤1 mm, and much smaller for both endemic and cosmopolitan taxa (Figure 17) (Additional file 2 contains a list of ordered taxonomic names for Figures 16 and 17).

## Discussion

### A comparative overview of the Hawaiian non-marine macroalgal flora

The 1,407 macroalgal samples collected during the Hawaiian Freshwater Algal Biodiversity Survey represent, by far, the most exhaustive survey of non-marine algae for a tropical oceanic island archipelago yet reported. Previous studies in the tropical Pacific have yielded between seven and 167 samples, and have resulted in algal checklists of 12–160 taxa [20],[42]-[47]. Algal studies of the islands of Yap, in the Caroline Islands, revealed a diverse flora of cyanobacteria, green algae, red algae, euglenoids and dinoflagellates totaling 63 taxa [42],[43], while, in contrast, a study of stream macroalgae of the islands of Fiji suggested an impoverished stream algal diversity of 15 taxa representing cyanobacteria, green algae and red algae [44]. Similar to the flora from Fiji, a rapid survey of fresh waters from the Austral Islands of French Polynesia yielded 12 species, representing cyanobacteria, green algae and xanthophytes [46].

A number of previous studies of Hawaiian stream algae have been conducted, which yielded from 34 taxa [20] to 42 taxa [45] to 160 taxa [47]; albeit all identifications from these past studies were morphologically based, and thus are not completely comparable to the current research, and were also more limited in collection intensity. For example, many of the common taxa previously reported from the Hawaiian Islands have been subsequently demonstrated through molecular analyses to represent different, but recognized species (e.g. *Cloniophora plumosa* from Hawaii is now recognized to be *C. spicata*[38]), endemic Hawaiian taxa (e.g. collections of the cyanobacterium *Nostochopsis*), or members of different genera (previous Hawaiian records of the green algal genus *Basicladia* appear to now be attributable to *Rhizoclonium*[48]; and this study. All previous previous records of the red alga *Audouinella* from freshwaters are now believed to represent sporophyte life history stages of the Batrachospermales and Thoreales in Hawaii [39]).

The present study is the first to integrate taxonomic collections from a multitude of non-marine algal habitats, including streams, wet walls, high elevation bogs, taro fields, ditches and flumes, cave walls and terrestrial areas for a tropical island algal flora. Two of these habitats were sufficiently well represented in the present survey collections to warrant individual treatment, and these have been published elsewhere as reports on the non-marine algae of Hawaiian taro fields [49] (which differ in being characterized by slow or absent water movement and higher temperatures than nearby streams) and high elevation bogs [50] (which are unique habitats in the Hawaiian Islands characterized by a high degree of isolation, low pH, high rainfall, and lower temperatures than many of the other sampled habitats).

### Molecular markers and example within-lineage patterns

A total of eight different molecular markers were employed in this study to characterize the non-marine algal diversity of the Hawaiian Islands. Due to the large scope of phylogenetic diversity encompassed in the collections (e.g. cyanobacteria as well as multiple eukaryotic algal lineages), we aimed to establish a multi-step molecular assessment that allowed initial broad-based characterization of all collections (based on the UPA marker), with subsequent use of one or more lineage-specific markers (e.g. 16S, COI, *tuf*A, ITS, LSU, *rbcL*[51]-[58]) for comparisons among closely related samples. Our previous biodiversity survey of the Hawaiian Rhodophyta (red algae [59],[60]) as well as other algal investigations [61]-[66] have provided support for the use of the UPA marker as a near-universal region of the plastid genome that enables construction of a molecular biodiversity framework for almost all algae, and we advocate for the use of this marker in multi-step molecular characterizations or analyses of environmental DNA samples where characterization of both cyanobacterial and eukaryotic algal diversity is desirable. Our second most widely applied marker, which spans the V4 region of nuclear SSU [67],[68], also resulted in generation of a large number of sequences, albeit fewer than UPA and of a more conserved nature than that marker (and also lacking in cyanobacterial representation). A comparison of the use of both markers for the characterization of environmental DNA from stream periphyton is underway (A. Carlile, R. Kodner & A. Sherwood, personal communication).

A number of taxa were well-represented in the survey, and the Hawaiian biodiversity represented by these lineages was characterized using a combination of the molecular approaches described above and microscopic analysis. For example, the charophycean genus *Spirogyra* has been noted as one of the most common freshwater macroalgae across the Hawaiian Islands in numerous publications dating back to 1876 [14]-[16],[69], and our survey collections of this genus spanned 86 locations on six islands, including diverse habitats such as streams, ditches, taro fields, bogs and ponds. The red algal “form genus” *Chantransia* was previously thought to be represented in the Hawaiian Islands by two lineages, *Sheathia arcuata* and *Nemalionopsis shawii*[70]; however, two additional taxa were added as a result of the survey collections (*Thorea hispida* and an undescribed *Thorea* sp.), doubling the known diversity of these freshwater red algal sporophytes in Hawaii [39]. Some non-marine representatives of the Cladophorales (*Cladophora* and *Rhizoclonium*), which can be difficult or impossible to identify using only morphological characters were demonstrated in some cases to be molecularly homogeneous, as demonstrated by others, despite substantial morphological variation (i.e. freshwater *Cladophora*[71]), and in other cases to harbor genus-level molecular diversity masked by a simple morphology (i.e. *Rhizoclonium*; A. Havens, A. Carlile & A. Sherwood, personal observations). Thus, one of the most valuable outcomes of the biodiversity survey approach of collecting and characterizing all non-marine macroalgae from a geographical region is that it allows multi-taxon large scale comparisons that can be compared to more accurately assess taxonomy and infer broader distributional patterns.

### Biogeographic patterns of the Hawaiian non-marine algae

Baas Becking’s ideas of microorganisms being globally distributed apply to organisms too small to see with the unaided eye, yet may be applicable to the study of freshwater macroalgae given that the dispersal forms of these organisms (spores, fragments, etc.) fall into the <1 mm size range (Figures 16 and 17). As a consequence, organism size may not predict distribution patterns well. Dispersal mechanisms of non-marine algae have been the subject of much speculation but little empirical research. A number of means of transport have been proposed, including air currents, vector-assisted transport (e.g. on the feet or feathers of birds, in the guts of fish or cases of aquatic invertebrates, via anthropogenic means), or rafting [72]-[74]. Although the initial colonization of the volcanically produced Hawaiian Islands by non-marine algae must have occurred by long-distance transport [4], dispersal among the islands likely played an increasingly important role in shaping the flora of the archipelago.

The non-marine algal flora of the Hawaiian Islands has almost certainly been impacted by humans since their first arrival in the Islands (between 400–1100 AD [2]). Polynesian introductions of numerous plant species for food and other uses may account for the first anthropogenic changes to the flora, but an acceleration of human impact over the past several hundred years (European contact in 1778 [2]) has probably had an even greater influence its composition, with spores and fragments being transported to and among the islands from activities such as the aquarium and ornamental plant trades, hiking, transport of building materials and heavy machinery, and farming. Any attempts to interpret algal biogeographic patterns must take these influences into consideration.

What is evident from the survey collection analyses is that no consistent biogeographic pattern emerges to explain the origins of the non-marine algal flora of the Hawaiian Islands. Some remarkably widespread taxa were characterized that are also known from many locations worldwide, including the clonally propogating red alga *Compsopogon caeruleus* and the green alga *Cladophora glomerata*; distribution of the former to Hawaii may be a result of the aquarium trade [39], while ubiquity in the latter case may be a result of either anthropogenic or non-anthropogenic dispersal. A few taxa were demonstrated to consist of multiple, cryptic lineages that likely represent intraspecific taxonomic variation, including *Sheathia arcuata* and some *Spirogyra* spp. A number of other taxa were found to have affinities with samples from a more limited number of other regions, but with little obvious biogeographical connection, for example, *Cloniophora spicata*, *Nemalionopsis shawii*, *Pithophora roettleri*, *Schizomeris leiblienii* and *Thorea hispida* ([39], this study). In the absence of well-populated reference frameworks of collections from numerous locations it seems likely that these distributions are a product of under-sampling rather than being representative of true biogeographic patterns. Conversely, several taxa were identified that are currently considered endemic to the Hawaiian Islands (e.g. *Kumanoa alakaiensis*, *K. spermatiophora*, *Nostochopsis* n.sp., *Scytonematopsis contorta*, *Thorea* n.sp.), and it is possible that expanded distributions of these taxa may be recognized in the future with increased sampling and characterization of the non-marine algal floras of other tropical Pacific Islands. It should also be noted that a number of microalgal taxa have also been recently described from the Hawaiian Islands as a result of this biodiversity survey, which serves to further emphasize the rich flora of this archipelago that is being illuminated through this opportunity for focused taxonomic attention [41],[75]-[77].

Accurate taxonomic identification of algae is not straightforward, and much depends on the species concept employed. Many of the taxa reported in this survey were described decades, if not centuries ago, and the formal descriptions of these taxa are anchored in morphological characters. While a great deal of information can be gleaned from macroscopic and microscopic observation, over the past two decades numerous examples of cryptic and pseudo-cryptic variation in algae have been highlighted through the analysis of molecular data [78],[79], and a definitive application of a pre-existing taxonomic name can only be made in light of demonstrated molecular affinity with type material; an exhausting and impossible prospect for the plethora of collections resulting from a biodiversity survey. Thus, it is expected that the taxonomic names applied to Hawaiian non-marine algae will be re-visited and re-examined as more comparative data become available. Finally, several practical difficulties played into a tradeoff between the number of samples that could be characterized and the number for which species-level taxonomic names could be applied. Most non-marine algal collections were relatively small in size (<2 cm in length) and were often heavily epiphytized; obtaining sufficient amounts of clean material for DNA extraction and PCR amplification was challenging. Additionally, many of the common taxa are typically assigned taxonomic names based heavily on characters pertaining to sexual reproduction (e.g. *Oedogonium*, *Mougeotia*, *Spirogyra*, *Zygnema*[80]-[83]), which was very infrequently observed in the field-collected samples. Attempts to induce sexual reproduction in culture in the laboratory were frequently unsuccessful; these observations suggest that sexual reproduction in Hawaiian non-marine algae may naturally occur much less frequently than for those in other geographical locations.

## Conclusions

The Hawaiian Freshwater Algal Biodiversity Survey yielded the largest sequence data compilation yet for tropical non-marine algae, which will serve as a baseline for comparison of new collections within the Hawaiian Islands and to other tropical regions. Key features of the biodiversity survey include the following: 1) The Hawaiian Freshwater Algal Database, which includes data for both collection sites and individual algal samples, which was designed and implemented as a key organizational component of the study, and serves as a web-accessible project portal to the scientific and broader communities. These kinds of digital resources are becoming increasingly recognized as critical data sources to those extracting and compiling broader scale patterns [84]. 2) Analysis of the 1,786 collecting sites and resultant 1,407 macroalgal samples, which informed the construction of the first comprehensive checklists for non-marine algae from unusual habitats in the Hawaiian Islands (taro fields and bogs [49],[50]), and an updated taxonomic checklist of Hawaiian non-marine macroalgae (Additional file 1). 3) In-depth assessment of common Hawaiian taxa, including representatives of the green algae (*Cloniophora*, *Spirogyra*, members of the Chaetophoraceae and Cladophoraceae), red algae (*Compsopogon*, *Chantransia* forms of *Nemalionopsis, Sheathia* and *Thorea*) and several cyanobacteria (e.g. *Nostoc* and *Nostochopsis*). Future research characterizing other tropical non-marine algal floras is needed to be able to draw comparisons between the flora of the Hawaiian Islands and elsewhere, to foster an understanding of the level of endemism in these isolated systems, and to illuminate the biodiversity of these understudied components of tropical island floras.

## Methods

### Sampling strategy and taxonomic scope

Samples were field-collected during five multi-island expeditions (July 2009, January 2010, May 2010, January 2011 and January 2012), and numerous smaller collecting trips to individual islands. Islands yielding substantial diversity were collected multiple times (Kauai, Oahu, Maui and Hawaii), while those with either limited diversity or a limited number of accessible habitats were collected only once (Molokai and Lanai). Expeditions were planned for different times of the year to capture some of the seasonality inherent in the wet-dry alternation of seasons in the Hawaiian Islands. All non-marine habitats were targeted, including streams, wet walls (drippy vertical surfaces that are not influenced by sea spray; typically associated with streams and waterfalls), high elevation bogs, taro fields (agricultural fields dedicated to cultivation of wetland taro plants), ditches and flumes (which were commonly constructed in the Hawaiian Islands for water transport for large-scale agriculture), lakes/reservoirs, cave walls and terrestrial areas (such as roadsides, damp ground, cement surfaces, etc.). Sampling sites were not selected randomly, but based on a combination of accessibility and likelihood of yielding interesting algae. Samples were collected from streams, bogs, taro fields, ditches and flumes using a glass-bottomed view box and long-handled forceps or a turkey-baster, while those from other habitats were collected by directly viewing the substratum and collecting the sample with forceps or scraping it with a spoon or razor blade. Samples were kept in WhirlPak™ bags until processed in the laboratory. All macroalgae (i.e. algae that could be seen with the naked eye, either as individuals or in colonial form) were targeted for this component of the survey, including both eukaryotic and cyanobacterial taxa. Some microalgal identifications were included when they were identified as part of a macroalgal collection (i.e. during surveys of taro field or high elevation bog habitats). Many more microalgal collections were made during the survey than are presented here – these will be published under separate taxonomic treatments by the co-authors of this study specializing in microalgal taxonomy. Each distinct site that was collected was given a five-digit “environmental accession” number, and the WhirlPak™ bag containing the algal material from that site was labeled with that number.

### Sample processing and analysis

Environmental samples were examined live with instructional-grade compound light microscopes (e.g. Wolfe B3 series) taken on inter-island collecting expeditions, and samples were divided so that diatoms, microscopic soft algae, and cyanobacteria could be studied in detail in the respective laboratories of various members of the collection team. The remainder of the environmental accession material, which included the macroalgae, was examined with a stereomicroscope (Zeiss SteREO Discovery v12) in the Sherwood laboratory on Oahu and individual taxa were cleaned and preserved for molecular study (by desiccating in silica gel or freezing at −20°C) and/or isolated for culturing. Environmental samples were initially placed in 60 × 15 mm petri dishes in a WC/L1 modified media, which consisted of a WC media base [85] and L1 trace metals solution [86]. Individual strains were isolated through serial dilution into either 24- or 96-well culture plates and subsequent micromanipulation into subcultures until unialgal cultures with enough material for molecular and morphological characterizations were obtained [87]. Cultures were maintained at room temperature (21-24°C) under approximately 100–250 μmol/sec/m^2^ PAR, conditions typical for those in Hawaiian freshwater systems where samples were collected [88],[89]. All remaining material was fixed in 2.5% CaCO_3_-buffered glutaraldehyde in 20-mL scintillation vials and is currently held (at 4°C) in the Sherwood laboratory at the University of Hawaii. Fixed material was examined with a compound light microscope (Olympus BX-41 or Zeiss AxioImager A1), and duplicate semi-permanent corn syrup microscope slides were made as vouchers. For a few of the larger taxa (e.g. Characeae), herbarium sheets were made. Morphological characters were compared to those in the phycological literature [80]-[83],[90],[91] and references within), and an identification to the lowest possible taxonomic level was assigned. In many cases the characters necessary for species-level (or, in some cases, even genus-level) identification were not present in the field-collected material (e.g., those pertaining to sexual reproduction, which was not commonly observed in the samples). For each identification, an accession number was assigned (the “isolate accession”), which consisted of ten digits – the first five of which comprised the environmental accession, and the second five of which indicated the number of the taxon isolated or identified (e.g. isolate accession 04380–00001 indicates the first taxon identified from environmental sample 04380).

DNA was extracted from field-collected and cultured material using a Qiagen DNeasy Plant Mini Kit. One primary aim of the project was to generate and compare baseline molecular marker sequences for as many taxa as possible, and thus one or two markers were sequenced for all or most samples (UPA and SSU), with additional markers selected based on available comparative data and the level of systematic investigation deemed necessary (see Table 1 for a list of primers used and references for PCR cycling conditions). All samples were amplified via PCR for the UPA marker [61],[92], while the partial SSU marker was subsequently added as a universal marker for all eukaryotic samples, and several additional markers (e.g. COI, *rbcL*, 16S rRNA, LSU, *tuf*A) were used for lineage-specific taxonomic or phylogeographic investigations. PCR product purification and sequencing followed Carlile & Sherwood [39]. Cyanobacterial 16S rRNA sequences were cloned (1–3 clones per taxon) following Vaccarino & Johansen [40]; clones are indicated in phylogenetic analyses with the suffix “a”, “b”, or “c” following the isolate accession.

**Table 1:** Primers and references for PCR amplification conditions of markers employed in the current study

**Marker**	**Source**	**Primers**	**Reference(s)**	**Taxa characterized**
16S rRNA	Cyanobacterial genome	Primer 1 (5′ CTC TGT GTG CCT AGG TAT CC 3′) and Primer 2 (5′ GGG GAA TTT TCC GCA ATG GG 3′)	[51]	Cyanobacteria
COI	Mitochondrial	GazF1 (5′ TCA ACA AAT CAT AAA GAT ATT GG 3′) and GazR1 (5′ ACT TCT GGA TGT CCA AAA AAY CA 3′)	[52]	Rhodophyta
ITS	Nuclear	CladoITS-9 F (5′ CCG CCC GTC GCT CCT ACC GAT TGG GTG TG 3′) and CladoITS7R (5′ TCC CTT TTC GCT CGC CGT TAC TA 3′)	[93]	Chlorophyta
LSU	Nuclear	C’1 (5′ ACC CGC TGA ATT TAA GCA TAT 3′) and D2 (5′ TCC GTG TTT CAA GAC GG 3′); C1FL (5' ACC CGC TGA ACT TAA GC 3′) and D2FL (5' GGT CCG TGT TTC AAG 3′)	[53],[54]	Chlorophyta
SSU	Nuclear	SR4 (5′ AGC CGC GGT AAT TCC AGC T 3′) and SR9 (5′ AAC TAA GAA CGG CAT GCA C 3′)	[94]	All eukaryotic lineages
*rbcL*	Plastid	F160 (5′ CCT CAA CCA GGA GTA GAT CC 3′) and rbcLR (5′ ACA TTT GCT GTT GGA GTC TC 3′) (Rhodophyta); Comp1 (5′ GAA TCT TCT ACA GCA ACT TGG AC 3′) and Comp 2 (5′ GCA TCT CTT ATT ATT TGA GGA CC 3′) (Rhodophyta); RH1 (5′ ATG TCA CCA CAA ACA GAA ACT AAA GC 3′) and rbcL-R1351-Zyg (5′ AGC AGC TAA TTC AGG ACT CAA 3′) (Streptophyta)	[55]-[57]	Rhodophyta and Streptophyta
*tuf*A	Plastid	tufAF (5′ TGA AAC AGA AMA WCG TCA TTA TGC 3′) and tufAR (5′ CCT TCN CGA ATM GCR AAW CGC 3′)	[58]	Streptophyta and Chlorophyta
UPA	Plastid	p23SrV_f1 (5′ GGA CAG AAA GAC CCT ATG AA 3′) and p23SrV_r1 (5′ TCA GCC TGT TAT CCC TAG AG 3′)	[61]	All lineages

Algal lineages characterized using each marker are listed.

### Data storage and dissemination

All associated sample and collection site data (photographs and micrographs, locality information, taxonomic identifications, DNA sequences, voucher type and archival information) were entered into the Hawaiian Freshwater Algal Database (H*fw*ADB), which was modeled after the Hawaiian Algal Database (HADB) and built specifically to organize and display the project data. H*fw*ADB is described in full [95] and is internet accessible at http://algae.manoa.hawaii.edu/hfwadb . H*fw*ADB holds images and other relevant data for both “environmental accessions” (i.e. georeferenced collection sites) and “isolate accessions” (i.e. individual algal identifications and their associated data, including micrographs and DNA sequence data).

### Data analyses

#### Mapping

Environmental accession (i.e. locality) data were downloaded from H*fw*ADB and plotted on a map of the Main Hawaiian Islands using GPS Visualizer ( http://www.gpsvisualizer.com/ ).

#### DNA barcoding

Sequences of each marker were downloaded from H*fw*ADB and aligned using Clustal X [96]. Neighbor-joining (NJ) trees for the 16S rRNA, COI, LSU, SSU, *rbcL*, and UPA markers were constructed using uncorrected p-distances in MEGA v. 5.05 [97] to examine patterns of molecular diversity. The NJ trees are presented as a visual display of the quantity and nucleotide variation in DNA sequences of Hawaiian non-marine algae. Although some authors are now electing to omit tree-based DNA barcode comparisons and present sequence divergences in tabular form [98], NJ trees are included here as a more easily interpretable display of the sequence data gathered and compared for this survey. Only a small number of closely-related taxa were sequenced for the ITS (n =5; *Cloniophora* and *Stigeoclonium*) and *tuf*A (n =7; *Cloniophora*) markers, hence summaries of those sequences are not shown here. Summary labels were added to the tree figures to indicate proposed taxonomic boundaries. Occasionally sequences were obtained that did not phylogenetically correspond to the intended taxon; in these cases the sequences were entered into H*fw*ADB with the designation “contaminant sequence”, and these are included in the DNA barcoding analyses as an additional indication of Hawaiian algal diversity.

#### Categories of distribution

Taxa determined to be well-characterized at the species level were further investigated for patterns of distribution. All taxa identified in the survey that were confidently identified to the level of species, and for which a species name could be applied, were queried through AlgaeBase ( www.algaebase.org ) [99] as well as the primary literature for distributional records. Distributions (presence/absence) were recorded by Hawaiian Island, and subsequently for each of the following broad geographic regions: Pacific Islands, Australia and New Zealand, Asia, North America, the Caribbean, South America, Europe, and Africa. To infer the degree of endemism versus cosmopolitanism for the Hawaiian freshwater algal flora, taxa were binned as belonging to one of four categories of distribution, and then compared to other members of the flora. The four categories employed were 1) those taxa reported from six or more regions outside of Hawaii (i.e. cosmopolitan, or approaching cosmopolitan in distribution), 2) 4–5 other regions, 3) 1–3 other regions, and 4) within-Hawaii only (i.e. putatively endemic to the Hawaiian Islands). Taxa classified into one of the four categories were further investigated in the literature to obtain thallus size range and “dispersal unit” (i.e., motile cells, gametes, fragments, etc.) size range; these values were graphically compared across distributional categories.

### Availability of supporting data

The data sets supporting the results of this article are available in the Dryad repository, doi:10.5061/dryad.ns1m7, and in GenBank [KM676560 - KM676564 for ITS, KM676565 - KM676567 for *tuf*A, KM676568 - KM676828 for UPA, KM676829 - KM676881 for LSU, KM676882 - KM677026 for SSU, KM677027 - KM677127 for *rbcL*].

## Competing interests

The authors declare that they have no competing interests.

## Authors’ contributions

ARS wrote the initial draft of the manuscript, co-conceived of the study, and participated in its design, execution and coordination. ALC, JMN and KYC participated in the field work and coordination of the study and generated the molecular data. JPK, JJ, RLL and GGP participated in the design, coordination and field work of the study. ARS, JPK, JJ, RLL and GGP obtained funding. All authors read and approved the final manuscript.

## Additional files

## Supplementary Material

Additional file 1 **Hawaiian non-marine algal checklist.** Checklist of non-marine algae collected and identified as part of the Hawaiian Freshwater Algal Biodiversity Survey. Click here for file

Additional file 2 **List of taxon names, in order, belonging to each distributional category illustrated in Figures** 16**and**17**.** Click here for file
