# Supplementary material for: The Hawaiian freshwater algae biodiversity survey (2009–2014): systematic and biogeographic trends with an emphasis on the macroalgae
Source: BMC Ecol. 2014 Oct 25;14:28. doi: 10.1186/s12898-014-0028-2 (PMC4222836; doi:10.1186/s12898-014-0028-2)
Supplement: Additional file 1 — Hawaiian non-marine algal checklist. Checklist of non-marine algae collected and identified as part of the Hawaiian Freshwater Algal Biodiversity Survey. [file s12898-014-0028-2-S1.docx]

**Additional File 1.** Checklist of non-marine algae collected and identified as part of the Hawaiian Freshwater Algal Biodiversity Survey.

| **Major lineage** | **Order** | **Family** | **Genus** | **Species (and subspecific taxon)** | **Authority** | **Islands^1^** | **Habitats^2^** |
| --- | --- | --- | --- | --- | --- | --- | --- |
| Streptophyta (Charophyceae) | Charales | Characeae | *Chara* | *braunii* | C.C. Gmelin | K, M | O, T |
| Streptophyta (Charophyceae) | Charales | Characeae | *Chara* | *zeylanica* | Willdenow | O | O |
| Streptophyta (Charophyceae) | Charales | Characeae | *Chara* | sp. |  | H | T |
| Streptophyta (Charophyceae) | Charales | Characeae | *Nitella* | sp. |  | O, Mo, M, H | T |
| Streptophyta (Charophyceae) | Klebsormidiales | Klebsormidiaceae | *Klebsormidium* | *flaccidum* | (Kützing) P.C.Silva, K.R.Mattox & W.H.Blackwell | K, O | TR |
| Streptophyta (Charophyceae) | Klebsormidiales | Klebsormidiaceae | *Klebsormidium* | sp. 1 |  | K, O, Mo, H | B, D, S, TR, W |
| Streptophyta (Charophyceae) | Klebsormidiales | Klebsormidiaceae | *Klebsormidium* | sp. 2 |  | O | S |
| Streptophyta (Charophyceae) | Coleochaetales | Coleochaetaceae | *Coleochaete* | *orbicularis* | Pringsheim | O | O |
| Streptophyta (Charophyceae) | Zygnematales | Closteriaceae | *Closterium* | *gracile* | Brébisson ex Ralfs | H | B |
| Streptophyta (Charophyceae) | Zygnematales | Closteriaceae | *Closterium* | *moniliferum* | Ehrenberg ex Ralfs | O | B |
| Streptophyta (Charophyceae) | Zygnematales | Closteriaceae | *Closterium* | *setaceum* | Ehrenberg ex Ralfs | H | S |
| Streptophyta (Charophyceae) | Zygnematales | Closteriaceae | *Closterium* | *striolatum* | Ehrenberg ex Ralfs | O | B |
| Streptophyta (Charophyceae) | Zygnematales | Closteriaceae | *Closterium* | sp. |  | K, O, Mo, L, M, H | B, S, TR |
| Streptophyta (Charophyceae) | Zygnematales | Closteriaceae | *Cosmarium* | *anceps* | P. Lundell | H | D |
| Streptophyta (Charophyceae) | Zygnematales | Closteriaceae | *Cosmarium* | *botrytis* | Meneghini ex Ralfs | H | D |
| Streptophyta (Charophyceae) | Zygnematales | Closteriaceae | *Cosmarium* | *caelatum* | Ralfs | H | D |
| Streptophyta (Charophyceae) | Zygnematales | Closteriaceae | *Cosmarium* | *obliquum* | Nordstedt | H | D |
| Streptophyta (Charophyceae) | Zygnematales | Closteriaceae | *Cosmarium* | *pachydermum* | P. Lundell | O | W |
| Streptophyta (Charophyceae) | Zygnematales | Closteriaceae | *Cosmarium* | *pyramidatum* | Brébisson ex Ralfs | H | D |
| Streptophyta (Charophyceae) | Zygnematales | Closteriaceae | *Cosmarium* | *regnellii* | Wille | O, Mo | B |
| Streptophyta (Charophyceae) | Zygnematales | Closteriaceae | *Cosmarium* | *subcucumis* | Schmidle | H | B, D |
| Streptophyta (Charophyceae) | Zygnematales | Closteriaceae | *Cosmarium* | sp. |  | K, O, Mo, M, H | B, L, O, S, T, W |
| Streptophyta (Charophyceae) | Zygnematales | Desmidiaceae | *Actinotaenium* | *cucurbita* | (Brébisson ex Ralfs) Teiling | H | B |
| Streptophyta (Charophyceae) | Zygnematales | Desmidiaceae | *Actinotaenium* | *diplosporum* | (P. Lundell) Teiling | H | D |
| Streptophyta (Charophyceae) | Zygnematales | Desmidiaceae | *Actinotaenium* | *diplosporum* var. *americanum* | (West & G.S.West) Teiling | H | B |
| Streptophyta (Charophyceae) | Zygnematales | Desmidiaceae | *Actinotaenium* | *cruciferum* | De Bary (Teiling) | H | D, S |
| Streptophyta (Charophyceae) | Zygnematales | Desmidiaceae | *Desmidium* | *aptogonum* | Brébisson ex Kützing | H | S |
| Streptophyta (Charophyceae) | Zygnematales | Desmidiaceae | *Desmidium* | sp. |  | M, H | B, S, T |
| Streptophyta (Charophyceae) | Zygnematales | Desmidiaceae | *Euastrum* | sp. |  | K, O, Mo, H | L, O, S, TR |
| Streptophyta (Charophyceae) | Zygnematales | Desmidiaceae | *Haplotaenium* | *minutum* | (Ralfs) Bando | H | B |
| Streptophyta (Charophyceae) | Zygnematales | Desmidiaceae | *Hyalotheca* | *dissiliens* | Brébisson ex Ralfs | M | S, T |
| Streptophyta (Charophyceae) | Zygnematales | Desmidiaceae | *Hyalotheca* | sp. |  | O | S |
| Streptophyta (CharophHyceae) | Zygnematales | Desmidiaceae | *Micrasterias* | *truncata* | Brébisson ex Ralfs | K | B |
| StreptophyHta (Charophyceae) | Zygnematales | Desmidiaceae | *Pleurotaenium* | *trabecula* | Nägeli | O, Mo | O, S |
| Streptophyta (Charophyceae) | Zygnematales | Desmidiaceae | *Staurastrum* | *muricatum* | Brébisson ex Ralfs | H | B |
| Streptophyta (Charophyceae) | Zygnematales | Desmidiaceae | *Staurastrum* | *trihedrale* | Wolle | H | B, D |
| Streptophyta (Charophyceae) | Zygnematales | Desmidiaceae | *Staurastrum* | sp. |  | K, O, Mo, H | B, D, O, S, W |
| Streptophyta (Charophyceae) | Zygnematales | Desmidiaceae | *Tetmemorus* | *brebissonii* | Ralfs | O, Mo, H | B, D |
| Streptophyta (Charophyceae) | Zygnematales | Desmidiaceae | *Tetmemorus* | *laevis* | Kützing ex Ralfs | K, O, H | B, D, O |
| Streptophyta (Charophyceae) | Zygnematales | Mesotaeniaceae | *Cylindrocystis* | *brebissonii* | (Ralfs) De Bary | O, H | B |
| Streptophyta (Charophyceae) | Zygnematales | Mesotaeniaceae | *Cylindrocystis* | *gracilis* | I. Hirn | O, H | B |
| Streptophyta (Charophyceae) | Zygnematales | Mesotaeniaceae | *Cylindrocystis* | sp. |  | O | B |
| Streptophyta (Charophyceae) | Zygnematales | Mesotaeniaceae | *Mesotaenium* | sp. |  | H | B |
| Streptophyta (Charophyceae) | Zygnematales | Mesotaeniaceae | *Netrium* | *digitus* | (Brébisson ex Ralfs) Itzigsohn & Rothe | Mo, H | B |
| Streptophyta (Charophyceae) | Zygnematales | Mesotaeniaceae | *Netrium* | *oblongum* | (De Bary) Lütkemüller | H | B |
| Streptophyta (Charophyceae) | Zygnematales | Mesotaeniaceae | *Netrium* | sp. |  | K, O, Mo, L, M, H | B, O, S, T, W |
| Streptophyta (Charophyceae) | Zygnematales | Mesotaeniaceae | *Spirotaenia* | *kirchneri* | Lütkemüller | H | B |
| Streptophyta (Charophyceae) | Zygnematales | Peniaceae | *Gonatozygon* | *kinahanii* | (W. Archer) Rabenhorst | K, O, H | O, S, TR |
| Streptophyta (Charophyceae) | Zygnematales | Peniaceae | *Penium* | sp. |  | K | S, W |
| Streptophyta (Charophyceae) | Zygnematales | Zygnemataceae | *Mougeotia* | *capucina* | C. Agardh | M | S |
| Streptophyta (Charophyceae) | Zygnematales | Zygnemataceae | *Mougeotia* | sp. |  | K, O, Mo, M, H | B, D, O, S, T, TR, W |
| Streptophyta (Charophyceae) | Zygnematales | Zygnemataceae | *Mougeotiopsis* | sp. |  | K | B |
| Streptophyta (Charophyceae) | Zygnematales | Zygnemataceae | *Spirogyra* | *maxima* | (Hassall) Wittrock | O | S, T |
| Streptophyta (Charophyceae) | Zygnematales | Zygnemataceae | *Spirogyra* | sp. |  | K, O, Mo, M, H | B, O, S, T, W |
| Streptophyta (Charophyceae) | Zygnematales | Zygnemataceae | *Zygnema* | sp. |  | K, O, Mo, M, H | B, O, S, T |
|  |  |  |  |  |  |  |  |
| Chlorophyta (Chlorophyceae) | Chaetophorales | Chaetophoraceae | *Chaetophora* | *elegans* | (Roth) C. Agardh | K, O | S |
| Chlorophyta (Chlorophyceae) | Chaetophorales | Chaetophoraceae | *Stigeoclonium* | sp. |  | K, O, Mo, M, H | D, L, S, T, W |
| Chlorophyta (Chlorophyceae) | Chaetophorales | Schizomeridaceae | *Schizomeris* | *leibleinii* | Kützing | O, H | S |
| Chlorophyta (Chlorophyceae) | Microsporales | Microsporaceae | *Microspora* | sp. |  | K, O, Mo, M, H | B, D, L, O, S, TR, W |
| Chlorophyta (Chlorophyceae) | Oedogoniales | Oedogoniaceae | *Bulbochaete* | sp. |  | K, M, H | S |
| Chlorophyta (Chlorophyceae) | Oedogoniales | Oedogoniaceae | *Oedogonium* | sp. |  | K, O, Mo, M, H | D, O, S, T, TR, W |
| Chlorophyta (Chlorophyceae) | Sphaeropleales | Hydrodictaceae | *Hydrodictyon* | *reticulatum* | (Linneaus) Bory de Saint Vincent | K, O | S, T |
| Chlorophyta (Chlorophyceae) | Sphaeropleales | Hydrodictaceae | *Pediastrum* | sp. |  | K, H | O, S |
| Chlorophyta (Chlorophyceae) | Sphaeropleales | Scenedesmaceae | *Coelastrum* | sp. |  | K | S |
| Chlorophyta (Chlorophyceae) | Sphaeropleales | Scenedesmaceae | *Desmodesmus* | sp. |  | K, O, H | L, W |
| Chlorophyta (Chlorophyceae) | Sphaeropleales | Scenedesmaceae | *Dimorphococcus* | sp. |  | H | S |
| Chlorophyta (Chlorophyceae) | Sphaeropleales | Scenedesmaceae | *Scenedesmus* | sp. |  | K, O, M, H | L, O, S, T, W |
| Chlorophyta (Chlorophyceae) | Sphaeropleales | Selenastraceae | *Ankistrodesmus* | sp. |  | M, K | S, T |
| Chlorophyta (Chlorophyceae) | Tetrasporales | Tetrasporaceae | *Tetraspora* | sp. |  | K, O, M, H | B, S |
| Chlorophyta (Chlorophyceae) | Volvocales | Chlamydomonadaceae | *Chlamydomonas* | sp. |  | K | S |
|  |  |  |  |  |  |  |  |
| Chlorophyta (Trebouxiophyceae) | Chlorellales | Chlorellaceae | *Dictyosphaerium* | sp. |  | H | B |
| Chlorophyta (Trebouxiophyceae) | Chlorellales | Chlorellaceae | *Geminella* | *minor* | (Nägeli) Heering | H | B |
| Chlorophyta (Trebouxiophyceae) | Chlorellales | Chlorellaceae | *Geminella* | sp. |  | H | S |
| Chlorophyta (Trebouxiophyceae) | Chlorellales | Chlorellaceae | *Mucidosphaerium* | *pulchellum* | (H.C.Wood) C.Bock, Proschold & Krienitz | H | B |
| Chlorophyta (Trebouxiophyceae) | Chlorellales | Chlorellaceae | *Zoochlorella* | sp. |  | M | S |
| Chlorophyta (Trebouxiophyceae) | Chlorellales | Oocystaceae | *Oocystis* | sp. |  | K | S |
| Chlorophyta (Trebouxiophyceae) | Microthamniales | Microthamniaceae | *Microthamnion* | *kuetzingianum* | Nägeli ex Kützing | K | B |
|  |  |  |  |  |  |  |  |
| Chlorophyta (Ulvophyceae) | Cladophorales | Cladophoraceae | *Aegagropila* | *linnaei* | Kützing | O | S |
| Chlorophyta (Ulvophyceae) | Cladophorales | Cladophoraceae | *Cladophora* | *glomerata* | (Linneaus) Kützing | K, O, M, H | D, S, T |
| Chlorophyta (Ulvophyceae) | Cladophorales | Cladophoraceae | *Pithophora* | *roettleri* | (Roth) Wittrock | K, O, M, H | D, O, S, T |
| Chlorophyta (Ulvophyceae) | Cladophorales | Cladophoraceae | *Rhizoclonium* | sp. |  | K, O, M, H | D, O, S, T, TR, W |
| Chlorophyta (Ulvophyceae) | Cladophorales | Cladophoraceae | *Rhizoclonium*-like new genus | sp. |  | K | TR |
| Chlorophyta (Ulvophyceae) | Ulvales | Cloniophoraceae | *Cloniophora* | *spicata* | (Schmidle) Islam | K, O, Mo, M, H | D, S, T |
| Chlorophyta (Ulvophyceae) | Trentepohliales | Trentepohliaceae | *Trentepohlia* | *abietina* | (Flotow) Hansgirg | H | T |
| Chlorophyta (Ulvophyceae) | Trentepohliales | Trentepohliaceae | *Trentepohlia* | *arborum* | (C. Agardh) Hariot | O, M, H | T |
| Chlorophyta (Ulvophyceae) | Trentepohliales | Trentepohliaceae | *Trentepohlia* | sp. |  | M, L, H | O, T |
| Chlorophyta (Ulvophyceae) | Ulotrichales | Ulotrichaceae | *Ulothrix* | sp. |  | K, M, H | S, W |
|  |  |  |  |  |  |  |  |
| Cyanobacteria | Chroococcales | Chroococcaceae | *Chroococcus* | *limneticus* | Lemmermann | K | TR |
| Cyanobacteria | Chroococcales | Hydrococcaceae | *Pleurocapsa* | *minor* | Hansgirg | O | W |
| Cyanobacteria | Chroococcales | Hydrococcaceae | *Pleurocapsa* | sp. |  | O | S |
| Cyanobacteria | Nostocales | Hapalosiphonaceae | *Fischerella* | sp. |  | K | TR |
| Cyanobacteria | Nostocales | Hapalosiphonaceae | *Nostochopsis* | sp. |  | K, O | S, T |
| Cyanobacteria | Nostocales | Microchaetaceae | *Camptylonemopsis* | sp. |  | O | S |
| Cyanobacteria | Nostocales | Microchaetaceae | *Fortiea* | sp. |  | O | S |
| Cyanobacteria | Nostocales | Microchaetaceae | *Petalonema* | sp. |  | O | S |
| Cyanobacteria | Nostocales | Microchaetaceae | *Tolypothrix* | sp. |  | O | S |
| Cyanobacteria | Nostocales | Nostocaceae | *Anabaena* | sp. |  | K, M, H | T |
| Cyanobacteria | Nostocales | Nostocaceae | *Cylindrospermum* | *muscicola* | Kützing ex Bornet & Flahault | O | T |
| Cyanobacteria | Nostocales | Nostocaceae | *Cylindrospermum* | sp. |  | K, M | T |
| Cyanobacteria | Nostocales | Nostocaceae | *Hydrocoryne* | *spongiosa* | Schwabe ex Bornet & Flahault | K | S |
| Cyanobacteria | Nostocales | Nostocaceae | *Nostoc* | *pruniforme* | C.Agardh ex Bornet & Flahault | O, M | S |
| Cyanobacteria | Nostocales | Nostocaceae | *Nostoc* | sp. |  | K, O, Mo, M, H | D, S, TR |
| Cyanobacteria | Nostocales | Rivulariaceae | *Calothrix* | *braunii* | Bornet & Flahault | M | S |
| Cyanobacteria | Nostocales | Rivulariaceae | *Calothrix* | sp. |  | K, O | S, T, TR |
| Cyanobacteria | Nostocales | Scytonemataceae | *Brasilonema* | *octagenarum* | R.Anguiar, M.F.Fiore, M.W.Franco, M.C.Ventrella, A.S.Lorenzi, C.A.Vanetti & A.C.Alfenas | O | TR |
| Cyanobacteria | Nostocales | Scytonemataceae | *Brasilonema* | sp. |  | O, M | S, TR |
| Cyanobacteria | Nostocales | Scytonemataceae | *Scytonema* | sp. |  | K, O | S, T, TR |
| Cyanobacteria | Nostocales | Scytonemataceae | *Scytonematopsis* | *contorta* | M.A.Vaccarino & J.R.Johansen | O | S, TR |
| Cyanobacteria | Nostocales | Stigonemataceae | *Stigonema* | sp. |  | O | TR |
| Cyanobacteria | Oscillatoriales | Oscillatoriaceae | *Homeothrix* | sp. |  | M | S |
| Cyanobacteria | Oscillatoriales | Oscillatoriaceae | *Lyngbya* | sp. |  | H | T |
| Cyanobacteria | Oscillatoriales | Oscillatoriaceae | *Oscillatoria* | *princeps* | Vaucher ex Gomont | M, H | T |
| Cyanobacteria | Oscillatoriales | Oscillatoriaceae | *Oscillatoria* | sp. |  | O | T |
| Cyanobacteria | Oscillatoriales | Phormidiaceae | *Phormidium* | *retzii* | Kützing ex Gomont | O, M | T |
| Cyanobacteria | Oscillatoriales | Phormidiaceae | *Phormidium* | sp. |  | M | S |
| Cyanobacteria | Oscillatoriales | Schizotricaceae | *Schizothrix* | *arenaria* | Gomont | O | O |
| Cyanobacteria | Synechococcales | Chamaesiphonaceae | *Chamaesiphon* | *aggregatus* | (Janczewski) Geitler | O | S |
| Cyanobacteria | Synechococcales | Synechococcaceae | *Synechococcus* | *elongatus* | (Nägeli) Nägeli | K | S |
|  |  |  |  |  |  |  |  |
| Euglenozoa | Euglenales | Euglenaceae | *Euglena* | sp. |  | K, O | B, O, S, T |
| Euglenozoa | Euglenales | Euglenaceae | *Phacus* | sp. |  | K | S |
|  |  |  |  |  |  |  |  |
| Chromista (Xanthophyceae) | Tribonematales | Tribonemataceae | *Tribonema* | *affine* | (Kützing) G.S.West | Mo, H | D, O, S |
| Chromista (Xanthophyceae) | Tribonematales | Tribonemataceae | *Tribonema* | sp. |  | K, Mo, H | D, S, TR |
| Chromista (Xanthophyceae) | Vaucheriales | Vaucheriaceae | *Vaucheria* | sp. |  | K, O, M, H | D, O, S, T, TR, W |
| Chromista (Bacillariophyceae) | Biddulphiales | Biddulphiaceae | *Hydrosera* | *whampoensis* | (A.F.Schwarz) Deby | K, O, Mo, M, H | D, S, T |
| Chromista (Bacillariophyceae) | Biddulphiales | Biddulphiaceae | *Terpsinoë* | *musica* | Ehrenberg | K, H | S |
| Chromista (Bacillariophyceae) | Melosirales | Melosiraceae | *Melosira* | *varians* | C.Agardh | O, M, H | S |
| Chromista (Bacillariophyceae) | Triceratiales | Triceratiaceae | *Pleurosira* | *laevis* | [(Ehrenberg) Compère](http://www.algaebase.org/search/species/detail/?species_id=y81946a1f29e45486&sk=0&from=results) | K, O, H | S, T |
|  |  |  |  |  |  |  |  |
| Rhodophyta | Batrachospermales | Batrachospermaceae | *Batrachospermum* | *arcuatum*^3^ | Kylin | K, O, Mo, M, H | D, S, T |
| Rhodophyta | Batrachospermales | Batrachospermaceae | *Kumanoa* | *alakaiensis* | A.R. Sherwood, C.A. Jones & K.Y. Conklin | K | S |
| Rhodophyta | Batrachospermales | Batrachospermaceae | *Kumanoa* | *spermatiophora* | (M.L.Vis & Sheath) Entwisle, M.L.Vis, W.B.Chiasson, Necchi & A.R.Sherwood | M | S |
| Rhodophyta | Compsopogonales | Compsopogonaceae | *Compsopogon* | *caeruleus* | [(Balbis ex C.Agardh) Montagne](http://www.algaebase.org/search/species/detail/?species_id=Sb6859c9516396463&sk=0&from=results) | K, O, M, H | D, S, T, TR |
| Rhodophyta | Hildenbrandiales | Hildenbrandiaceae | *Hildenbrandia* | *angolensis* | Welwitsch ex West & G.S.West | O, M | S |
| Rhodophyta | Thoreales | Thoreaceae | *Nemalionopsis* | *shawii*^3^ | Skuja | M | S |
| Rhodophyta | Thoreales | Thoreaceae | *Thorea* | *hispida*^3^ | (Thore) Desvaux | O | S |
| Rhodophyta | Thoreales | Thoreaceae | *Thorea* | sp.^3^ |  | K | S |

^1^Island(s) of distribution, from northwest to southeast: K = Kauai, O = Oahu, M = Maui, Mo = Molokai, L = Lanai, H = Hawaii

^2^Habitat(s): B = bog, D = ditch, L = lake, O = other (pond, puddle, seasonal marsh, etc.), S = stream, T = taro field, TR = terrestrial, W = wet wall

^3^as *Chantransia* phase only (sporophyte alternative life-history phase)
