# Supplementary material for: The Hawaiian freshwater algae biodiversity survey (2009–2014): systematic and biogeographic trends with an emphasis on the macroalgae
Source: BMC Ecol. 2014 Oct 25;14:28. doi: 10.1186/s12898-014-0028-2 (PMC4222836; doi:10.1186/s12898-014-0028-2)
Supplement: Additional file 2 — List of taxon names, in order, belonging to each distributional category illustrated in Figures 16and17. [file s12898-014-0028-2-S2.docx]

**Additional File 2.** List of taxon names, in order, belonging to each distributional category illustrated in Figs. 16 and 17.

**Figure 16 order of names, listed from top to bottom:**

**Category 4 (endemic)** – *Nostochopsis* n. sp., *Scytonematopsis contorta*, *Thorea* n. sp., *Kumanoa alakaiensis*, *Kumanoa spermatiophora*

**Category 3** – *Hydrosera whampoensis*, *Chamaesiphon aggregatus*, *Terpsinoe musica*, *Brasilonema octagenarum*, *Nemalionopsis shawii*, *Thorea hispida*, *Hydrocoryne spongiosa*, *Mougeotia capucina*, *Schizomeris leibleinii*, *Nostoc pruniforme*

**Category 2** – *Pleurocapsa minor*, *Chroococcus limneticus*, *Melosira varians*, *Pleurosira laevis*, *Calothrix braunii*, *Geminella minor*, *Nostoc commune*, *Sheathia arcuata*, *Chaetophora elegans*, *Cylindrospermum musica*, *Aegagropila linnaei*, *Pithophora roettleri*, *Hydrodictyon reticulatum*, *Klebsormidium flaccidum*, *Tribonema affine*, *Chara braunii*, *Chara zeylanica*

**Category 1 (cosmopolitan)** – *Microthamnion kuetzingianum*, *Mucidophaerium pulchellum*, *Trentepohlia abietina*, *Hildenbrandia angolensis*, *Coleochaete orbicularis*, *Schizothrix arenaria*, *Oscillatoria princeps*, *Phormidium retzii*, *Cloniophora spicata*, *Trentepohlia arborum*, *Cladophora glomerata*, *Compsopogon caeruleus*

**Figure 17 order of names, listed from top to bottom:**

**Category 4 (endemic)** – *Nostochopsis* n. sp., *Scytonematopsis contorta*, *Kumanoa alakaiensis*, *Kumanoa spermatiophora*, *Thorea* n. sp.

**Category 3** – *Hydrocoryne spongiosa*, *Chamaesiphon aggregatus*, *Schizomeris leibleinii*, *Nostoc pruniforme*, *Hydrosera whampoensis*, *Nemalionopsis shawii*, *Thorea hispida*, *Brasilonema octagenarum*, *Mougeotia capucina*, *Terpsinoe musica*

**Category 2** – *Pleurocapsa minor*, *Nostoc commune*, *Tribonema affine*, *Calothrix braunii*, *Sheathia arcuata*, *Chaetophora elegans*, *Hydrodictyon reticulatum*, *Klebsormidium flaccidum*, *Chroococcus limneticus*, *Cylindrospermum musicola*, *Geminella minor*, *Melosira varians*, *Chara braunii*, *Chara zeylanica*, *Pleurosira laevis*, *Pithophora roettleri*, *Aegagropila linnaei*

**Category 1 (cosmopolitan)** – *Oscillatoria princeps*, *Phormidium retzii*, *Mucidosphaerium pulchellum*, *Cloniophora spicata*, *Microthamnion kuetzingianum*, *Schizothrix arenaria*, *Coleochaete orbicularis*, *Cladophora glomerata*, *Trentepohlia abietina*, *Compsopogon caeruleus*, *Trentepohlia arborum*, *Hildenbrandia angolensis*
